# Experiential learning for primary healthcare workers in low- and middle-income countries: a scoping review protocol

**DOI:** 10.1186/s13643-019-1040-6

**Published:** 2019-05-20

**Authors:** Nkosinothando Chamane, Desmond Kuupiel, Tivani Phosa Mashamba-Thompson

**Affiliations:** 1 Discipline of Public Health Medicine, School of Nursing and Public Health, University of KwaZulu-Natal, Durban, South Africa; 2 Research for Sustainable Development Consult, Sunyani, Ghana

**Keywords:** Primary health care, Experiential learning, Low- and middle-income countries

## Abstract

**Background:**

Improving the quality of primary healthcare services is one of the global health priorities. Literature shows that the incompetency of healthcare providers has the potential to negatively affect the quality of the services provided. Experiential learning is one of the educational models that can be used to help improve healthcare service delivery. The main objective of this study is to systematically map literature on the evidence of experiential learning for primary healthcare workers in low- and middle-income countries (LMICs).

**Methods:**

This systematic scoping review’s search strategy will involve the following electronic databases: PubMed, Google Scholar, EBSOhost (Academic search complete, Health Source: Nursing/Academic Addition, MEDLINE) and open access for unpublished theses and dissertations. Websites such as the World Health Organization (WHO) and the departments of health website will be searched for policies and guidelines on experiential learning training programs. Following title searching, two-independent reviewers will conduct screening of abstracts and full articles. The screenings will be guided by the eligibility criteria. Data will be extracted from the included studies and the emerging themes will be analysed. The review team will analyse the implications of the findings in relation to the research question and aim of the study. The Mixed Method Appraisal Tool (MMAT) will be employed for quality appraisal of included studies.

**Discussion:**

We anticipate finding a significant number of studies on the applications of experiential learning in resource-limited settings. Findings will be disseminated through publication in a peer-reviewed journal, peer presentations as well as presentations at relevant conferences.

## Background

Provision of quality health care services for all is a global health priority [9]. Since the 1978 Alma-Ata Declaration, primary health care (PHC) is considered the main strategy to achieve health for all and to ensure universality, quality, equity, efficiency and sustainability of essential services [12]. PHC healthcare workers, mainly nurses, are the main custodians of PHC in low- and middle-income countries (LMICs). PHC nurses in LMICs practice in a poor resource and constantly changing environments. The changes include new and expanding roles for health professionals, increasing technological advances in treatment and care, new equipment and upscaling of rapid tests [3]. The competency of the user has been found to affect the reliability of the results produced using a technology device [10]. Incompetency of PHC providers can potentially affect the quality of services provided.

One of the key targets of the Sustainable Development Goals (SDGs 3.8) seeks to provide access to safe, effective, quality and affordable essential medicines and vaccines for all. Achievement of this goal is dependent on the competency of health care professionals [14]. Furthermore, competency of professionals is linked to the achievement of SDG 4, which concerns issues of quality education for all and promotion of lifelong learning. Lifelong learning for professionals can be achieved through continuous professional development (CPD) programs [13]. CPD is an essential characteristic of a profession and lasts throughout the individual’s personal and professional experience, thereby embracing the concept of lifelong learning [3]. Experiential learning is one of the educational models that can be used to achieve CPD and to improve health service delivery [1].

Many definitions of experiential learning can be found in literature, but in its simplest form, experiential learning is defined as learning achieved through the appropriate use of current experience. Many educational models based on experiential learning have been designed and used successfully as a method of training in continuing education in medicine and in many university courses intended for health professionals [1, 7]. However, the level of evidence on experiential learning training for primary healthcare settings in LMICs is not known. The main objective of this review study is to map available evidence on experiential learning training programs for PHC workers in LMICs. We anticipate that this study will provide information that will help improve the understanding of experiential learning, highlight knowledge gaps and stimulate future research.

## Methodology

We will conduct a scoping review to map evidence on experiential learning programs for PHC workers in LMICs. This study protocol has not been registered in PROSPERO because it is a scoping review. This scoping review will be guided by the Arksey and O’Malley scoping review framework stipulating the following steps [2]:

1. Identifying the research question
2. Identifying relevant studies
3. Study selection
4. Charting the data
5. Collating, summarising and reporting the results

Quality assessment of each of the included primary studies will be done as per [6] recommendations [6].

### Identifying the research question

The research question of interest is: what evidence is available on experiential learning for PHC workers in LMICs?

Sub-questions are as follows:

- What is the utility of experiential learning approaches for PHC workers?
- What is the acceptability of experiential learning for PHC workers?
- What is the effectiveness of experiential learning approach on quality service delivery at PHC level?

### Eligibility criteria

The Population Concept Context (PCC) framework has been used to determine the eligibility of the research question, as illustrated in Table 1.

**Table 1:** PCC framework

Criteria	Determinants
Population	Primary health care workers (all category of nurses, councillors and student nurses) in LMICs
Concept	Experiential learning training programs (onsite training, field-based experiences and participatory learning)
Context	Quality improvement

### Identifying relevant studies

This scoping review will include all study designs, peer-reviewed papers in published journals as well as grey literature such as unpublished theses, documents from departmental websites among others. To identify relevant studies, an electronic search will be conducted in the following databases: PubMed, Google Scholar, EBSCOhost (Academic search complete, Health Source: Nursing/Academic Addition, MEDLINE) and open access for unpublished theses and dissertations. Websites such as the World Health Organization (WHO) and the departments of health websites will be searched for policies and guidelines for experiential learning training programs. We will search literature published in any language from inception to the most current search. A further search of relevant studies will also be conducted through the ‘Cited by’ search and through screening of citations included in the reference lists of included articles. The search keywords will include Primary health care and experiential learning. We will utilise Boolean terms (AND, OR) to separate the keywords. MeSH (Medical Subject Headings) terms will also be included during the search. The search results will be imported to endnote library and duplicates will be removed, and the selected studies will be screened against the eligibility criteria. The study search strategy using the study keywords was piloted to determine the feasibility of conducting this study, and the results obtained are illustrated in Table 2.

**Table 2:** Pilot database search results

Keyword search	Date of search	Search engine used	No. of publications retrieved
(“primary health care”[MeSH Terms] OR (“primary”[All Fields] AND “health”[All Fields] AND “care”[All Fields]) OR “primary health care”[All Fields]) AND (“problem-based learning”[MeSH Terms] OR (“problem-based”[All Fields] AND “learning”[All Fields]) OR “problem-based learning”[All Fields] OR (“experiential”[All Fields] AND “learning”[All Fields]) OR “experiential learning”[All Fields])	2018-01-16	PubMed	476

### Study selection

An eligibility criterion was developed to ensure that selected studies contain relevant information to answer this review research question as follows:

#### The inclusion criteria

We will include studies that present evidence on:

- Primary health care workers: frontline nurses, councillors and nursing students
- Experiential learning programs in primary health care
- Onsite/in-house training programs in primary health care
- Studies conducted in LMICs
- Studies published from inception to 2018

#### Exclusion criteria

The scoping review will exclude:

- ■ Studies that present evidence of experiential learning on other professionals such as teachers.
- ■ Studies conducted in high-income countries.

The primary investigator will conduct a comprehensive search and screening of the study titles from the above-mentioned databases. All studies with eligible titles will be exported to an Endnote X8 library specifically created for this review. All duplicates will be removed before abstract screening. An abstract screening tool will be developed using google forms and sent to two reviewers, including the primary investigator for abstract screening. The reviewers will conduct abstract screening followed by full article screening of selected studies independently, with reference to the eligibility criteria. Articles which do not contain abstracts will be passed onto full article screening. We will contact authors to request for studies that are not retrievable from the above-mentioned databases. Any discrepancies in reviewers’ results at abstract screening will be resolved by discussion until consensus is reached, before progressing to full article screening. Discrepancies at full article screening will be resolved by involving a third reviewer. The University of KwaZulu-Natal (UKZN) library services will be utilised to optimise our study search strategy to help with retrieving and finding included full articles which may not be accessible from the above-mentioned databases. The screening results will be reported using an adapted Preferred Reporting Items for Systematic Reviews and Meta-Analyses (PRISMA) chart.

### Data charting

A data extraction tool will be developed using google forms to extract and process relevant information to answer the review study’s research question: what evidence is available on experiential learning for ensuring PHC quality service delivery in LMICs? The data extraction tool as illustrated in Table 3 will be validated by involving a second reviewer in the extraction of the first ten included studies and will continually be updated.

**Table 3:** Data charting form

Author and date	
Journal full reference	
Aims or research questions	
Study population	
Geographical setting	
Study design	
Theoretical background	
Experiential learning as an intervention	
Professional level of the trainer	
Experiential learning outcomes	
Most relevant findings	
Most significant findings	
Level of evidence	
Conclusions	
Comments	

### Quality appraisal

An electronic version of Mixed Method Appraisal Tool (MMAT) will be adapted and developed using google forms to assess the quality of the included primary studies [11]. Two reviewers will be involved in quality appraisal of included primary studies. The MMAT allows for quality appraisal and description of methodological quality for three methodological domains: mixed methods, qualitative and quantitative (further subdivided into three sub-domains: randomised controlled, nonrandomized, and descriptive). The quality of each of the selected studies will be assessed according to the relevant methodological domain in the tool. The MMAT will also be used to examine the appropriateness of study aims, the context relevance, theoretical inferences to answer research questions, author’ discussions and conclusions. The overall quality for each of the included studies will be calculated by following the MMAT guidelines (score = number of criteria met/total score in each domain) and results presented using the following descriptors:

- ▶ Low quality (25%), where minimal criteria is met
- ▶ Average (50%)
- ▶ Above average (75%)
- ▶ High quality (100%), where all criteria is met

For mixed methods studies, the premise is that the overall quality of a combination cannot be more than the quality of its weakest component. Thus, the overall quality score will be the lowest score of the study components (qualitative or quantitative) [11].

### Collating, summarising and reporting the results

The findings of this systematic scoping review will be analysed using the content analysis approach of themes emerging from relevant and significant findings. Emerging themes will be analysed, critically examining how they contribute to answering the research question. The review team will meet to discuss findings, resolve discrepancies and finalise results. The review team will also analyse the implications of the findings in relation to the aim of the study, future research and evidential framework for policy and application in LMICs.

## Discussion

Experiential learning is a type of learning centred on the active participation of individuals in a field. When implemented well, it can afford PHC workers in resource-limited setting opportunities of continuous development they are limited from [5]. A significant number of primary studies have been conducted on the applications of this intervention to health education. [8] has demonstrated that using participatory based learning programs such as experiential learning can strengthen community research partnerships as well as provide valuable training experiences for current and aspiring health personnel [8]. In addition, [4] found that the reflective writing step of experiential learning can contribute to identifying and addressing of gaps in medical education for resource-limited settings [4].

The scoping review will exclude studies conducted in high-income countries, as well as those conducted on PHC workers in private institutions. These studies will be excluded because of the differences in setting and resource limitations, which are not the same as those of most PHC clinics in LMICs.

To the best of our knowledge, no systematic reviews or scoping review studies on experiential learning in LMICs have been conducted in the past. Conducting the proposed scoping review will contribute to research through providing the status of knowledge available on the evidence of this intervention. This may also influence policymaking and designing of context-specific experiential learning models to ensure continual service delivery and ultimately improve health outcomes. The findings of this scoping review will be disseminated through publication in a peer-reviewed journal, conference presentations as well as through presentations to relevant stakeholders.

## Abbreviations

CPD: Continuous professional development
LMICS: Low- and middle-income countries
PHC: Primary health care

## Authors’ information

Nkosinothando Chamane: Currently a PhD student in the Discipline of Public Health Medicine, School of Nursing and Public Health, University of Kwazulu-Natal, South Africa.

Dr Desmond Kuupiel: He is a Postdoctoral Researcher in the Discipline of Public Health Medicine, School of Nursing and Public Health, University of Kwazulu-Natal, South Africa.

Dr Tivani Phosa Mashamba-Thompson: She is a Senior Lecturer and the Academic Leader (Research) for the School of Nursing and Public Health, University of KwaZulu-Natal, South Africa.
